# Assessment of primary care physicians' knowledge of chronic kidney disease in Poland

**DOI:** 10.3389/fpubh.2022.1032240

**Published:** 2022-10-20

**Authors:** Alicja Jazienicka-Kiełb, Mateusz Babicki, Magdalena Krajewska, Andrzej Oko, Karolina Kłoda, Agnieszka Mastalerz-Migas

**Affiliations:** ^1^Department of Family Medicine, Wroclaw Medical University, Wrocław, Poland; ^2^Department of Nephrology and Transplantation Medicine, Wroclaw Medical University, Wrocław, Poland; ^3^Department of Nephrology, Transplantology and Internal Diseases, Poznan University of Medical Sciences, Poznan, Poland; ^4^MEDFIT KAROLINA KŁODA, Szczecin, Poland

**Keywords:** chronic kidney disease (CKD), knowledge, physicians, family medicine, general practice

## Abstract

Chronic kidney disease (CKD) affects 10–15% of the adult population worldwide and is a major societal problem. A latent course of the disease and little alarming, gradually increasing symptoms usually do not cause concern in patients and diagnostic vigilance in physicians. CKD is most often diagnosed in its end-stage when treatment options are extremely limited. This study aims to assess the knowledge of CKD among primary care physicians (PCPs) in Poland. A CAWI survey was conducted based on an authors' own questionnaire that consisted of two parts. The first part concerned patients' socioeconomic data while the second part consisted of nine single- and multiple-choice questions assessing knowledge of the criterion for diagnosis, risk factors, diagnostic evaluation, and course of CKD. A total of 610 physicians took part in the survey, including 502 (82.3%) who fully completed the questionnaire. Women accounted for 83.1% of the study group. The mean age of the study group was 37.4 ± 10.1 years. Specialists or resident physicians in family medicine accounted for 79.9% of respondents and 93.8% of physicians are those who mainly work in primary care settings. In the knowledge test, the mean score obtained by physicians was 6.5 ± 1.3 out of possible 9, with only 2.4% of respondents answering all questions correctly. According to the survey, 78.4% of respondents correctly indicated the criterion for the diagnosis of CKD, while only 68.9% identified a test for increased urinary albumin loss as the one of the greatest diagnostic values in the early stages of CKD. More than half, 63.1%, of physicians selected the correct set of answers in the multiple-choice question regarding CKD risk factors. Despite a fairly high level of knowledge among family medicine physicians regarding the causes, risk factors and course of CKD, there is a need for further education and an increase in the factual information held by this professional group, especially that the vast majority of PCPs declare a desire to expand their knowledge and believe that this will help them in their daily clinical practice.

## Introduction

Chronic kidney disease (CKD) is a multifactorial condition, resulting from a reduced number of nephrons in response to an ongoing inflammatory process. Along with diabetes, hypertension and other cardiovascular diseases, CKD is one of the diseases of affluence in the 21st century (1). Data concerning the epidemiology of CKD in Poland are scarce and there are no up-to-date statistics on how many patients suffer from the disease. According to the 2007 PolNef study, the largest epidemiological study of CKD in Poland, the disease was diagnosed in 11.9% of patients after including albuminuria as a diagnostic criterion. With additional analysis of urine sediment and renal ultrasound changes, the criterion for the diagnosis of CKD was met by 18% of patients (1–3). The NATPOL 2011 study, a nationwide analysis of the prevalence and control of heart disease risk factors in Poland, found CKD in 5.8% of patients who participated in that study and, according to the results, estimated the prevalence of CKD to be almost 2 million in the Polish population aged 18–79 years (4). According to available global data, it is estimated that CKD may occur in up to 15% of the population (4–6).Consequently, CKD should be suspected in an even larger number of Polish people —up to 4 million.

According to official statistics provided by the Polish National Health Fund—NFZ, 210,000 Polish people have been diagnosed with CKD. It should be borne in mind, however, that reported cases usually concern the kidney failure, when patients remain under the constant care of nephrologists and are selected for renal replacement therapy (RRT) (7). In comparison with the previously cited data, this shows that the detection of CKD in Poland is underestimated.

The reason may be the lack of adequate awareness among patients and physicians regarding the causes, symptoms, risk factors, diagnostic evaluation, and treatment of CKD. This may contribute to a lack of adequate vigilance among physicians.

Many patients are only diagnosed in advanced stages of the disease, when alarming clinical signs appear and the only treatment offered is RRT. It was found that non-pharmacological management, when implemented early enough, can significantly reduce disease progression, prolong patients' lives, and improve their quality of life (1). This is why 1) diagnostic vigilance—when a patient visits the doctor's office for other chronic diseases that often coexist with CKD, 2) appropriate frequency of follow-up examinations and 3) knowledge of risk factors are of so much importance.

Given these aspects, PCPs' knowledge of CKD is crucial in the diagnostic and therapeutic process of CKD.

To the best knowledge of the authors, there are no Polish studies concerning the level of knowledge of CKD among PCPs. Moreover, there are also single references to this subject in the world literature, from countries such as the United States, Nigeria, Pakistan and Cameroon (8–11).

At the same time, in the United Kingdom (UK) where CKD is diagnosed and treated primarily in primary care settings, a tool was created in 2014—a questionnaire assessing confidence and knowledge in terms of the care of CKD patients compared to other chronic diseases. The tool was named QICKD-CCQ (Quality Improvement Interventions in Chronic Kidney Disease -The Clinician Confidence and Knowledge Questionnaire). Although the questionnaire met its expectations in a study concerning its practical use and the authors recommend this tool be added to the standard armamentarium of tools useful for PCPs, the study itself had several limitations. Such limitations included, for example, conducting the study not in practices selected at random but those indicated by researchers. Therefore, they were not representative of all family medicine physicians' practices in the UK (12).

Accordingly, the authors aimed to assess the level of knowledge of CKD among PCPs in Poland and determine the extent to which knowledge of this disease needs to be improved.

## Methodology

This study is a CAWI (Computer-Assisted Web Interview) survey using an authors' own questionnaire that was made available as part of the ankieta.pchn.edu.pl domain, which was created for the “Chronic Kidney Disease” project implemented by the Polish Society of Family Medicine and the Polish Society of Nephrology. Distribution of the questionnaire took place *via* facebook.com within the doctors' group, where membership is verified by medical license number. Furthermore, the questionnaire was distributed by e-mail using the mailing database of the Polish Society of Family Medicine. The target group of the study was physicians working in primary care settings. The survey distribution period was from 22 Feb. 2022 to 16 May 2022.

Prior to participating in the survey, respondents were informed of the aims and nature of the study. Subsequently, they gave their informed consent to participate in the study. During course of the study, its participants were allowed to withdraw from it without giving any reason. Participation was fully anonymous and voluntary, and respondents received no financial consideration for completing the survey.

The used author's own questionnaire consisted of two parts. The first part included questions assessing sociodemographic status (age, gender). Subsequently, data concerning professional status were collected, including the main place of work (primary care clinic/specialist outpatient clinic/hospital), its location (rural area/town < 50,000 inhabitants / city of 50,000–250,000 inhabitants / city > 250,000 inhabitants), years of seniority, career stage (specialist in family medicine / resident physician in family medicine / specialist in another specialty / resident physician in another specialty) and number of hours worked per week (≤10 h/11–24 h/ ≥25 h) in primary care settings. Using a 10-point Likert scale, respondents were asked to rate their level of knowledge regarding CKD.

The final stage of the survey concerned the level of knowledge of CKD. It consisted of both single- and multiple-choice questions. Within these questions, respondents were asked about the criterion for the diagnosis of CKD, the most common cause of CKD, diagnostic evaluation and clinical signs of CKD, and the most common cause of death in the course of CKD. Further multiple-choice questions addressed risk factors, preventive management during the early stage of CKD, and an assessment of cases when to be vigilant in terms of estimating eGFR. In each question, the maximum number of points was 1. In the case of single-answer questions, the respondent earned 1 point for each correct answer. For multiple-answer questions, 1 point was obtained for all indications of all correct answers. The maximum possible number of available points to score was 9. Final questions addressed the desire to improve knowledge of CKD. An English-language version of the survey is presented as supplementary material.

The study was conducted in accordance with the Declaration of Helsinki and approval was obtained from the Bioethics Committee at the Lower Silesian Medical Chamber; Resolution No. 1/BNR/2022.

The survey represents the first stage of a nationwide epidemiological and educational study concerning CKD. The project was designed in collaboration with the Polish Society of Family Medicine and the Polish Society of Nephrology. It is intended that the project will have three stages. The first stage will assess physicians' knowledge of CKD. The next stage involves conducting a voluntary, free, online educational course for all interested physicians. The final stage is a nationwide epidemiological study of CKD in a group at high risk of developing the disease. The project is ongoing and further publications of its results are planned for the future.

### Statistical analysis

The statistical analysis was conducted using Statistica software, version 13.0, StatSoft. The variables analyzed were qualitative, quantitative and ordinal. The Shapiro-Wilk test was used for assessing the normality of the distribution. Basic descriptive statistics were used for describing the study group and assessing the level of knowledge. Basic linear models were used for assessing the relationship between mean scores and gender, place of work, career stage, number of hours worked in primary care settings. In contrast, the Pearson's correlation was used for assessing the correlation between scores and age, years of seniority, subjective assessment of the level of knowledge regarding CKD. The effect of career stage and place of work on the distribution of answers to individual questions was assessed using the Pearson's chi-square test Statistical significance level was established at *p* < 0.05 for each case.

## Results

### Characteristics of the study group

A total of 610 physicians took part in the study, including 502 (82.6%) who completed the questionnaire. All respondents agreed to participate in the study. The vast majority of participants were women-417 (83.1%). The mean age of the study group was 37.4 ± 10.1 years (min. 24; max. 80). According to the survey, 401 (79.9%) physicians are specialists or resident physicians in family medicine and 471 (93.8%) physicians mainly work in primary care settings. The average seniority was 8.4 ± 8.9 years (min. 1, max. 51). A detailed description of the study group is shown in Table 1.

**Table 1:** Characteristics of the study group.

**Variable**		***N* (%)**
Gender	Male	82 (16.9)
	Female	417 (83.1)
	**Age [M ±SD]**	37.4 ± 10.1
Career stage	Has not begun specialist training	25 (5.0)
	Resident physician in family medicine	226 (45.0)
	Specialist in family medicine	175 (34.9)
	Resident physician in another specialty	23 (4.6)
	Specialist in another specialty	53 (10.5)
Place of work	City > 250,000 inhabitants	208 (41.4)
	City of 50,000–250,000 inhabitants	99 (19.8)
	Town < 50,000 inhabitants	115 (22.9)
	Rural areas	80 (15.9)
**Years of seniority [M ±SD]**		8.4 ± 8.9
Main place of work	Primary health care	471 (93.8)
	Outpatient health care (OHC)	6 (1.2)
	Hospital	25 (5.0)
Number of working hours in primary care settings [hours/week]	≥ 25 11–24 ≤ 10	412 (82.1) 67 (13.3) 23 (4.6)
Contact with CKD at work	Yes	489 (97.4)
	No	13 (2.6)

M, mean; SD, standard deviation.

### Level of knowledge regarding CKD

Based on a 10-point Likert Scale, the mean score assessing the subjective level of knowledge about CKD was 5.6 ± 1.66. In the knowledge test, the mean score obtained by physicians was 6.5 ± 1.3. Only 12 (2.4%) physicians answered all questions correctly. In contrast, the question-by-question analysis found that 394 (78.4%) physicians correctly identified the diagnostic criterion for CKD and 473 (94.2%) correctly identified the most common cause of the disease. Only 346 (68.9%) physicians correctly identified a test for urinary albumin loss as the one of greatest diagnostic value in the early stages of CKD. The most frequently indicated risk factors by physicians include diabetes (98.4%) and hypertension (96.8%); however, the correct set of risk factors was identified by only 317 (63.1%) physicians. A detailed comparison of all answers is shown in Table 2.

**Table 2:** The comparison of answers to questions assessing the level of knowledge of CKD.

**Question**		***N* (%)**
**Criterion for diagnosis of CKD**	**GFR < 60 ml/min./1.73m**^**2**^ **for at least 3 months**	394 (78.4)
	GFR < 90 ml/min./1.73m^2^ for at least 3 months	67 (13.3)
	GFR < 60 ml/min./1.73m^2^ for at least 1 month	23 (4.6)
	GFR < 90 ml/min./1.73m^2^ for at least 6 months	18 (3.7)
**Most common cause of CKD**	**Diabetic kidney disease**	473 (94.2)
	Coronary artery disease	13 (2.6)
	Chronic dehydration	13 (2.6)
	Polycystic kidney degeneration	3 (0.6)
	Neoplastic diseases of the urinary tract	0 (0.0)
**Can CKD be asymptomatic?**	**Yes, it can. Clinical signs of CKD develop slowly and become a concern to patients in the late stage of the disease**.	500 (99.6)
	Yes, it can. However, clinical signs of CKD appear early and are usually severe.	0 (0.0)
	No, it cannot. Clinical signs appear almost immediately.	2 (0.4)
**Which of the following tests is of the greatest diagnostic value in the early stages of CKD?**	**Test for increased urinary albumin loss**	346 (68.9)
	Serum urea levels	83 (16.5)
	Abnormal urine specific gravity	61 (12.2)
	Presence of erythrocytes in urine sediment	11 (2.2)
	White blood cell (WBC) count	1 (0.2)
**GFR at which RRT should be initiated**	**< 10 [ml/min./1.73 m** ^**2**^ **]**	436 (86.9)
	< 30 [ml/min./1.73 m^2^]	66 (13.1)
**Main cause of death in the course of CKD**	**Cardiovascular complications**	404 (80.5)
	Ketone coma	8 (1.6)
	Protein-calorie malnutrition	19 (3.8)
	Electrolyte imbalance	44 (8.7)
	Infections	27 (5.4)
**Risk factors for the development of CKD include (multiple-choice question):**	**Diabetes**	496 (98.8)
	**Hypertension**	486 (96.8)
	**Old age**	451 (89.4)
	**History of cardiovascular diseases**	445 (88.6)
	**Obesity**	359 (71.5)
	Regular physical activity	2 (0.4)
	**Percentage of correct answers**	317 (63.1)
Management during the early stage of CKD should include (multiple-choice answer):	**Proper treatment of underlying disease**	500 (99.6)
	**Avoidance of nephrotoxic drugs**	478 (95.2)
	**Reduction of dietary sodium intake**	403 (80.3)
	Introduction of a protein-rich diet	37 (7.4)
	Increase in dietary phosphate intake	9 (1.8)
	Admission to dialysis as soon as possible	3 (0.6)
	**Percentage of correct answers**	356 (70.9)
In whose patients should caution be exercised when estimating GFR?	**In patients with abnormal amounts of muscle tissue or with skeletal muscle diseases**	469 (93.4)
	**In obese patients**	355 (70.7)
	**In patients aged > 60 years**	303 (60.4)
	**In smokers**	71 (14.1)
	**Percentage of correct answers**	55 (11.0)
**Number of correct answers [M ±SD]**		6.5 ± 1.3

M, mean; SD, standard deviation. Correct answers are in bold.

**Figure 1:**
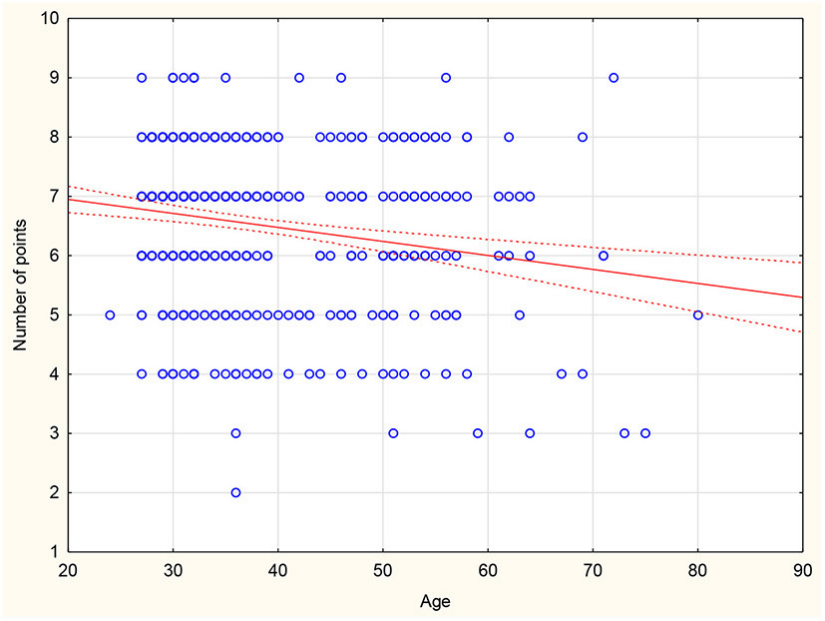
Correlation between age and scores on the CKD knowledge assessment test.

**Figure 2:**
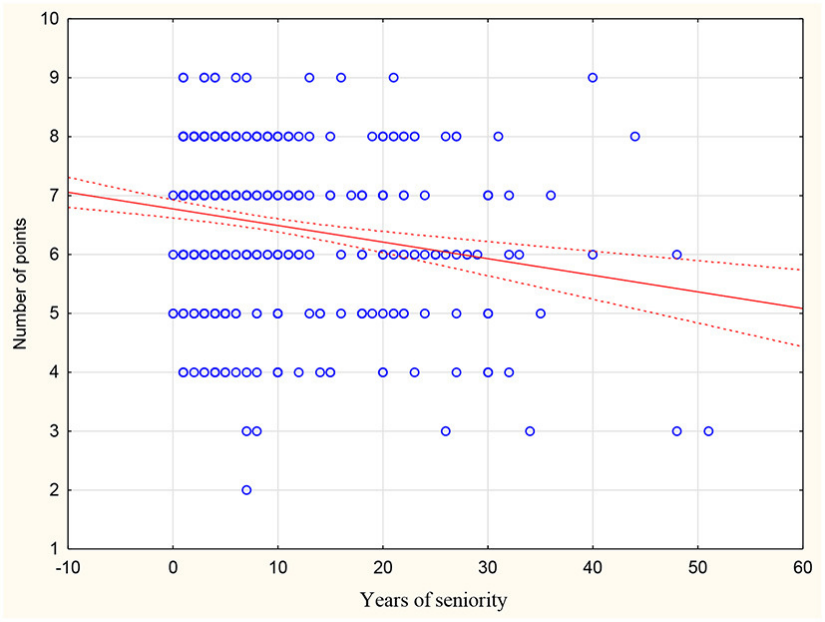
Correlation between years of seniority and scores on the CKD knowledge assessment test.

**Figure 3:**
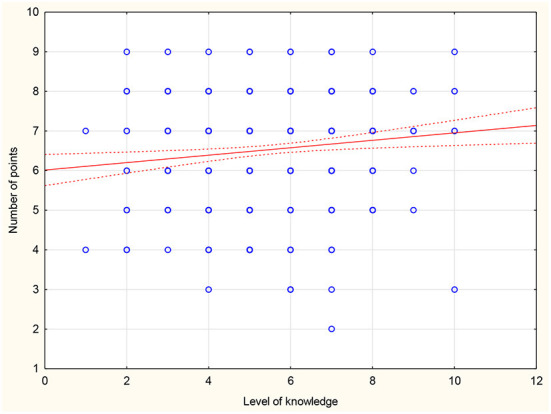
Correlation between level of knowledge and scores on the CKD knowledge assessment test.

There was no statistically significant difference between the mean scores compared to gender, career stage, place of work or number of hours worked in primary care settings. Those who declare contact with CKD scored on average 1.02 points higher than doctors who declare no contact with CKD in daily practice (*p* = 0.004). Moreover, an inverse correlation was found between respondents' age and mean score (r = −0.183; *p* < 0.001) or years of seniority (r = −0.194; *p* < 0.001) (Figures 1, 2). There was also a positive correlation between the subjective assessment of the level of knowledge and the mean score (r = 0.127; *p* = 0.007) (Figure 3). A detailed comparison of mean scores is shown in Table 3.

**Table 3:** The comparison of mean scores according to gender, career stage, place of work and past contact with CKD.

**Variable**		**Mean**	**B**	**SD**	**t**	**p**
Gender	Male	6.78	0.144	0.08	1.88	0.059
	Female	6.49	Ref.	Ref.	Ref.	Ref.
Career stage	Has not begun specialist training	6.24	−0.227	0.22	−1.05	0.295
	Resident physician in family medicine	6.72	Ref.	Ref.	Ref.	Ref.
	Specialist in family medicine	6.38	−0.084	0.11	−0.74	0.461
	Resident physician in another specialty	6.65	−0.185	0.22	−0.82	0.411
	Specialist in another specialty	6.34	−0.127	0.16	−0.79	0.430
Place of work	City > 250,000 inhabitants	6.57	0.043	0.09	0.49	0.623
	City of 50,000–250,000 inhabitants	6.48	−0.048	0.11	−0.44	0.661
	Town < 50,000 inhabitants	6.43	−0.098	0.11	−0.94	0.348
	Rural areas	6.64	Ref.	Ref.	Ref.	Ref.
Number of working hours in primary care settings [hours/week]	≥ 25	6.58	0.189	0.11	1.70	0.091
	11–24	6.37	0.017	0.14	0.12	0.902
	≤ 10	6.21	Ref.	Ref.	Ref.	Ref.
Contact with CKD at work	Yes	6.56	0.512	0.18	2.84	0.004
	No	5.54	Ref.	Ref.	Ref.	Ref.

Ref., reference; SD, standard deviation; B, coefficient value of a given variable; t, test value; p, statistical significance.

Furthermore, the analysis of individual questions in terms of the career stage revealed that resident physicians in family medicine were most likely to indicate the correct answer regarding the test of greatest diagnostic value in the early stages of CKD. The remaining questions revealed no statistically significant differences in terms of place of work and career stage. A detailed comparison is shown in Table 4.

**Table 4:** The comparison of individual questions according to career stage and place of work.

	**Career stage**						**Place of work**				
	**Percentage of correct answers n (%)**					**p**	**Percentage of correct answers** ***n*** **(%)**				**p**
	**Resident physician in family medicine**	**Specialist in family medicine**	**Resident physician in another specialty**	**Specialist in another specialty**	**Physician who has not begun specialist training**		**City > 250,000 inhabitants**	**City of 50,000 – 250,000 inhabitants**	**Town < 50,000 inhabitants**	**Rural areas**	
Criterion for diagnosis of CKD	184 (81.4)	137 (78.3)	18 (78.3)	38 (71.7)	17 (68.0)	0.376	160 (76.9)	82 (82.8)	85 (73.9)	67 (83.8)	0.246
Most common cause of CKD	215 (95.1)	164 (93.7)	22 (95.7)	50 (94.3)	22 (88.0)	0.682	195 (93.8)	93 (93.9)	108 (93.9)	77 (96.3)	0.867
Symptoms of CKD	225 (99.6)	174 (99.4)	23 (100.0)	53 (100.0)	25 (100.0)	0.968	208 (100.0)	98 (98.9)	115 (100)	79 (98.8)	0.297
Test with the greatest diagnostic value	173 (76.6)	107 (61.1)	16 (69.6)	36 (67.9)	14 (56.0)	**0.011**	140 (67.3)	68 (68.7)	80 (69.6)	58 (72.5)	0.856
GFR criterion for RRT	198 (87.6)	155 (88.6)	21 (91.3)	42 (79.3)	20 (80.0)	0.322	184 (88.5)	87 (87.9)	94 (81.7)	71 (88.8)	0.327
Cause of death	188 (83.2)	131 (74.9)	21 (91.3)	46 (86.8)	18 (72.0)	0.067	169 (80.8)	73 (73.7)	94 (81.7)	69 (86.3)	0.196
Risk factors	147 (65.0)	106 (60.6)	16 (69.6)	29 (54.7)	19 (76.0)	0.325	136 (65.4)	63 (63.6)	72 (62.6)	46 (57.5)	0.665
Management during the early stage of CKD	166 (73.5)	124 (70.9)	14 (60.9)	35 (66.0)	14 (60.9)	0.636	151 (72.6)	66 (66.7)	80 (69.6)	59 (73.8)	0.667
Caution when estimating GFR	23 (10.2)	19 (10.9)	2 (8.7)	7 (13.2)	4 (16.0)	0.879	26 (12.5)	12 (12.1)	12 (10.4)	5 (6.3)	0.476

Significant values are in bold with the significance level set at p < 0.05.

According to the study, 469 (93.4%) physicians agree or strongly agree after completing the survey that they intend to improve their level of knowledge regarding CKD in the near future. On the other hand, 496 (98.8%) physicians believe that this knowledge could be useful in their daily medical practice.

## Discussion

The role of the family medicine physician is crucial in terms of initiating diagnostic and therapeutic management at the appropriate stage of CKD, as well as in terms of monitoring the patient's health status and assessing the effectiveness of the treatment process. Family medicine physicians have more regular interaction with a patient than physicians in other specialities. During consultations for infectious diseases, chronic diseases and even during prescription consultations, family medicine physicians have the opportunity to identify risk factors for CKD in their patient and initiate diagnostic evaluation even in the latent stage of the disease (13). The control of the treatment process and the assessment of disease progression by the PCP is also very important due to the long waiting time for consultation in specialists in nephrology. According to available data, there are 1,389 nephrology physicians who are professionally active in Poland, and this number is assessed as insufficient (14, 15). Therefore, part of the responsibility in providing care for CKD patients should belong to general practitioners. However, a high level of awareness of the disease among physicians is necessary to provide adequate care.

The results of this study revealed knowledge gaps among PCPs in Poland and areas for potential educational intervention. PCPs are aware that their knowledge of CKD is not sufficiently comprehensive—in their subjective assessment of own knowledge, they gave themselves a score of 5.6 ± 1.66 on a ten-point Likert scale. Moreover, there was a positive correlation between the respondents' subjective assessment of their knowledge level and the knowledge test score obtained by them in the survey. This indicates the physicians' self-awareness regarding sophistication of their own knowledge and their understanding of the associated limitations. Furthermore, it should be noted that 93.4% of physicians agree with the statement that they intend to increase their level of knowledge of CKD in the near future. Almost 99% of respondents identify as true the statement that this knowledge could be useful in their daily clinical practice. The above-mentioned results clearly indicate a great need for educational activities, which are likely to be of interest to physicians working in primary care settings. This is in line with further intentions of the Chronic Kidney Disease Programme to provide a free educational course for interested physicians. A US study found an online course to be effective in improving knowledge of CKD among resident physicians in internal medicine, highlighting such advantages of online education as ease of access and use (16).

Physicians who declared that they had no interaction with CKD patients scored worse than their colleagues who were actively involved in providing care for these patients. CKD is a condition which is so prevalent and with so many risk factors that every PCP can expect to identify this disease in their patients. According to the authors, in terms of a declared lack of interaction with CKD patients it is more likely that physicians are insufficiently aware of the prevalence of this disease than that they actually have no contact with it. It should be noted that CKD is estimated to affect more than one in ten adult patients in Poland, so its prevalence is high (2). Only 78.4% of respondents identified a correct diagnostic criterion for CKD. This is a relatively low percentage, given that lack of knowledge of the definition and criterion for diagnosis (in this case it is the criterion based on eGFR) prevents identification of the disease in many cases and reduces diagnostic vigilance. In a similar survey conducted in Cameroon, the correct definition of CKD was indicated by only 58.8% of physicians (10). In other developing countries, results obtained from surveys assessing physicians' knowledge of the definition of CKD are even lower—only 38.8% of respondents from West Africa correctly defined CKD while only 38% of physicians from Pakistan were aware that GFR could be used for identifying CKD (8, 9). Furthermore, when asked about a laboratory test of particularly high value in the early stages of CKD, albuminuria was indicated by only 68.9% of respondents. Several reasons are possible for such low awareness of the value of this test. Albuminuria is not part of reimbursed services in primary care settings in Poland, making it much less used in daily practice. Similar results were obtained in other global studies—many physicians are unaware of the value of this test, which is one of the earliest indicators of kidney damage. Out of US resident physicians surveyed, not even half of them were aware that persistent albuminuria for 3 months allows the diagnosis of CKD. Albuminuria may be present and alert for further diagnostic evaluation when the results of other tests are within normal limits (1, 8, 9, 11, 16–18).

Diabetes and hypertension were identified by the vast majority of respondents as risk factors for CKD (98.8 and 96.8%, respectively). Similar data were obtained in other previous studies concerning knowledge of CKD among PCPs and internal medicine physicians. In terms of US respondents, 99% identified diabetes and hypertension as risk factors for CKD, while the above-mentioned disease entities were identified as risk factors by more than 80% of Pakistani respondents (diabetes 88.4%, hypertension 80%) (9, 11, 17). The risk factor of old age was also identified by the vast majority of respondents (89.4%). This factor was often overlooked by physicians among the answers selected during surveys in other countries. It was identified by only 33.6% of physicians in Karachi and 71% in the United States (8, 17). The elderly are particularly predisposed to CKD due to structural changes in the renal vasculature and a decrease in the number of active glomeruli. Deterioration of renal function is associated with aging and a decline in GFR starts as early as 40 years of age (2). Relatively few respondents identified obesity as a risk factor for CKD—only 71.5%. This answer was selected less frequently than diabetes and hypertension also among US resident physicians—only 38% of the physicians identified obesity as a risk factor for CKD (11). This is particularly alarming given that there has been a pandemic of obesity over the last 50 years (19, 20). Obesity is also one of the most common risk factors for CKD in children and adolescents (21). A complete set of all risk factors for CKD in the multiple-choice question was identified by only 63.1% of the physicians participating in this survey, which indicates a significant knowledge gap in this area and calls for a quick intervention to raise awareness among physicians.

In addition to diagnostic evaluation and risk assessment, the family medicine physician's role is to implement appropriate therapeutic management. This is particularly important in the early stages of CKD, when morphological changes are small, and appropriate management can help to inhibit the disease progression. In the question assessing the management during the early stage of the disease, 70.9% of respondents correctly indicated all the listed correct principles for the management during the early stage of CKD (proper treatment of underlying disease; avoidance of nephrotoxic drugs; reduction of dietary sodium intake). However, the authors are aware that these are not all the recommendations that should be followed by the CKD patient. The analysis of individual answers reveals that 99.6% of surveyed physicians indicated the need to treat underlying disease. This is crucial because, as it is well-known, CKD is most often a secondary condition of, among other things, diabetes and hypertension (1, 2). The awareness of family medicine physicians and their recommendations for behavioral intervention are of great value in terms of slowing the disease progression (13).

By analyzing the sum of correct answers obtained, an inverse correlation was found between the age of respondents and mean score obtained (r = −0.183; *p* < 0.001) as well as between years of seniority and mean score obtained (r = −0.194; *p* < 0.001). Resident physicians in family medicine scored highest on average in the knowledge test. These results are in line with those obtained in other studies, where resident physicians exhibited a higher level of knowledge regarding CKD compared to specialists (22). Simultaneously, the inverse relationship between the age of respondents and their score obtained is noteworthy. This relationship indicates the need for conducting training and educational programmes because as years of seniority increase, knowledge that is not updated can result in a decline in the quality of patient care.

In the US study, physicians (97% of respondents had been in the profession for more than 10 years) were asked about the difficulties associated with providing appropriate care to CKD patients. The most important obstacles that were pointed out by them included the lack of sufficient knowledge of CKD, the lack of clear guidelines for patient management and the difficulty in keeping up with dynamically changing recommendations. The respondents also pointed to the lack of a simple algorithm that would be useful in daily practice (23). The Australian team of Manski-Nankervis is working on such an algorithm. In 2021, the team published their proposal and the status of their work on a computer programme that is a sort of platform being tested within two family medicine physicians' practices. The programme is designed to assist in the identification of CKD, record-keeping, and continued patient management. Both family medicine physicians and specialists in nephrology—from academic and non-academic circles—are involved in building the platforms, as well as computer scientists, statisticians, lawyers and economists (24). The project of the authors of this study also uses a multidisciplinary approach that has the best chance of success in terms of increasing the rate of diagnosis and improving the quality of care for CKD. In China, attention is also being paid to the growing need for eHealth services for CKD. This need comes from both patients and physicians (25).The observations described above again support the need for education among PCPs in Poland. Specialists in family medicine who are burdened with work often do not have enough time and adequate knowledge of educational tools that are appropriate to their needs and may additionally fall into a routine in terms of their daily professional duties. Resident physicians are partly motivated to educate themselves on an ongoing basis and stay up to date with guidelines through the specialty training programme. As physicians get older, both mobility and willingness to use online educational courses often decrease due to their professional and family commitments. It would also be advisable to create educational tools for specialists in family medicine, so that they can easily and conveniently stay up to date with current recommendations and guidelines despite their busy schedules. The creation of a management algorithm would be useful in daily clinical practice, especially that US studies indicate the difficulty in terms of determining the appropriate timing for a family medicine physician to refer a patient to a specialist in nephrology (23, 26). There is also little knowledge among US physicians regarding drug dosage in patients with a history of CKD (27). According to studies conducted in the United States and Australia, knowledge of one's own disease is also very low among patients who suffer from CKD, which poses even greater challenges for the physician who provides care for them (28–30). Such a physician should communicate to the patient, in a clear and accessible way, the principles to be followed in their everyday life with the new disease.

The authors are aware of the limitation of this study, which is undoubtedly the lack of survey methodology—authors' own questions concerning CKD were used. To the best knowledge of the authors, however, there is no current tool validated under Polish conditions that could be used. The proposal for the tool originated in the UK but has several limitations (12). The authors are aware that these 10 questions addressing CKD are not sufficient to assess knowledge of the disease. The authors, however, attempted to select questions in such a way that they addressed different stages of the diagnostic and therapeutic process and were varied as possible. The creation of the questions was consulted with the specialists in nephrology who were patrons of the authors' project. Another limitation is the selection of the study group that is not representative of PCPs in Poland due to low age of respondents and significant predominance of women. For the reasons described above, the following results should not be considered reflective of the population as a whole, and further observations are necessary.

## Conclusions

In conclusion, this study reveals that the level of physicians' knowledge regarding CKD in Poland is insufficient, as a mean score for correct answers was 6.5 out of possible 9. Only three quarters of physicians know correct criteria for the diagnosis of CKD, 68.9% correctly indicate the diagnostic significance of albuminuria and 71.5% correctly select all risk factors for the disease. Moreover, 70.9% of the surveyed physicians correctly identify proper recommendations for the management during the early stage of CKD. The number of correct answers decreases with the work experience and age of the respondents. All this points to a lack of adequate awareness regarding CKD among physicians.

Therefore, there is a need to organize an appropriate educational offer, including e-learning, especially that physicians are motivated to use it. The educational offer should not only be addressed to resident physicians but also to specialists, who find it difficult to keep up with changing guidelines and recommendations in the course of their work and with their busy schedules. It should be noted that PCPs are highly motivated to educate themselves and expand their knowledge of CKD as declared in the questionnaire survey.

## Data availability statement

The raw data supporting the conclusions of this article will be made available by the authors, without undue reservation.

## Ethics statement

The studies involving human participants were reviewed and approved by the study was conducted in accordance with the Declaration of Helsinki and approval was obtained from the Bioethics Committee at the Lower Silesian Medical Chamber; Resolution No. 1/BNR/2022. The patients/participants provided their written informed consent to participate in this study.

## Author contributions

Conceptualization and data curation: AJ-K, MB, and AM-M. Formal analysis: MB. Funding acquisition and project administration: AM-M. Investigation: AJ-K and MB. Methodology and writing—review and editing: AJ-K, MB, MK, AO, KK, and AM-M. Supervision and validation: MK, AO, KK, and AM-M. Visualization: AJ-K, MB, and KK. Writing—original draft: AJ-K, MB, KK, and AM-M. All authors have read and agreed to the published version of the manuscript.

## Funding

The project was funded by the scientific activities of the Polish Society of Family Medicine.

## Conflict of interest

The authors declare that the research was conducted in the absence of any commercial or financial relationships that could be construed as a potential conflict of interest.

## Publisher's note

All claims expressed in this article are solely those of the authors and do not necessarily represent those of their affiliated organizations, or those of the publisher, the editors and the reviewers. Any product that may be evaluated in this article, or claim that may be made by its manufacturer, is not guaranteed or endorsed by the publisher.
